# Gemcitabine and cisplatin regimen facilitates prognosis of advanced nasopharyngeal carcinoma

**DOI:** 10.1002/cam4.1575

**Published:** 2018-05-23

**Authors:** Qiongxuan Li, Zhi Yin, Tingting Wang, Lizhang Chen, Zhanzhan Li

**Affiliations:** ^1^ Department of Epidemiology and Health Statistics Xiangya School of Public Health Central South University Changsha China; ^2^ Department of Oncology Xingya Hospital Central South University Changsha China

**Keywords:** chemotherapy, meta‐analysis, nasopharyngeal carcinoma, randomized controlled trial

## Abstract

This study was conducted to assess the efficacy and adverse effects of GP (gemcitabine + cisplatin) regimen and FP (fluouracil + cisplatin) regimen in treatment of advanced nasopharyngeal carcinoma. Systematic online searches were performed in PubMed, Web of Sciences, China Knowledge Infrastructure and Weipu from the inception to November 15, 2017. Potential studies were assessed using the Cochrane risk of bias scale. Statistical analyses were performed on Stata 14.0 and RevMan 5.3. Finally, twelve studies entered final qualitative synthesis and quantitative analysis. The GP regimen compared with the FP regimen had significantly higher 1‐year survival rate (relative risk (RR) = 1.07, 95% confidence interval (CI): 1.01‐1.13), significantly better performance in the fixed‐effect model (RR = 1.16, 95%CI: 1.04‐1.30) and significantly higher remission rate (RR = 1.17, 95%CI: 1.05‐1.29). Significant differences between regimens were found in gastrointestinal effects (RR = 0.58, 95%CI: 0.45‐0.74). No significant differences between regimens were found in reduced hemoglobin rate (RR = 0.55, 95%CI: 0.36‐1.21), neutropenia (RR = 1.84, 95%CI: 0.93‐5.02), or reduced platelet (RR = 1.25, 95%CI: 0.85‐1.75) and mucosal inflammation (RR = 0.81, 95%CI: 0.57‐1.16). Sensitivity analysis indicated the results remained stable. The funnel plot indicated some publication bias. In conclusion, the GP regimen outperforms the FP regimen in treatment of advanced nasopharyngeal carcinoma with no difference in adverse effects. We may consider the GP regimen a better choice, but this conclusion should be confirmed by high‐quality trials.

## 1 INTRODUCTION

Nasopharyngeal carcinoma (NPC) is an Epstein‐Barr virus‐associated epithelial malignancy and shows unusual disparity in ethnic and geographical distributions.^1^ This typical regional disease mostly occurs in South China and Southeast Asia with the incidence ranging from 30 to 50 per 10 000.^2^, ^3^ The incidence and morality of NPC in these areas rank first worldwide, indicating NPC has become an important public health issue in these areas. NPC has high malignant degree and is prone to lymph node metastasis and distant metastasis without obvious symptoms at early stage. NPC patients usually have been in the middle and late stage when confirmed. Reception of operation treatment is quite difficult for NPC patients because of the special anatomy.^4^ In clinical practice, radiotherapy is the primary choice for NPC patients at early stage, with the partly remission rate more than 90%. Currently, the primary reasons of treatment failure are distant metastasis, local recurrence, and regional lymph nodes, which account for 60%‐70%, ~20%, and ~16% of failures, respectively.^5^, ^6^ These situations severely affect the survival rate. Therefore, it is urgent to improve the survival rate and life quality of advanced NPC patients.

The combined treatment of radiotherapy and chemotherapy is the main way for advanced NPC. As is well‐known, the combination of fluorouracil and cisplatin is the classic first‐line treatment plan for advanced NPC, which has been supported mainly by randomized controlled trials, but rarely by evidence‐based medicine research.^7^ Studies show gemcitabine combined with cisplatin has a considerable therapeutic effect on advanced NPC, but the findings remain inconsistent because of differences in sample sizes, pathology types, and stages. In the present meta‐analysis, we systematically searched studies about treatment of advanced NPC and compared the curative effects and adverse reactions between 2 therapeutic regimens, aiming to provide more scientific and reliable evidences/guidelines for clinical treatment of NPC.

## 2 MATERIALS AND METHODS

Ethical approval was not applicable for this meta‐analysis based on previous studies. We referred to the *Preferred Reporting Items for Systematic Reviews and Meta‐Analysis* (PRISMA) guidelines.^8^

### 2.1 Literature search

Systematic online searches were performed in PubMed, Embase, Web of Sciences, China Knowledge Infrastructure and Weipu database from the inception to November 15, 2017. The following medical subject headings and keywords were used: advanced nasopharyngeal carcinoma, gemcitabine, fluorouracil, cisplatin, chemotherapy, randomized controlled trial or RCT. The specific usages were as follows: Step 1: “nasopharyngeal carcinoma”/[Topic] OR “NPC”/[Topic]; Step 2; “gemcitabine”/[Topic] OR “fluorouracil”/[Topic] OR “cisplatin”/[Topic]/“chemotherapy”/[Topic] AND results from Step 1. The references of relevant studies were also reviewed to identify potential studies. Languages were restricted to Chinese and English.

### 2.2 Inclusion and exclusion criteria

Two investigators independently conducted the literature search and initial screening, including removing duplicates, scanning titles and abstracts, and identifying records. The inclusion criteria were: (1) Study design: randomized controlled trial; (2) Study population: patients with locally advanced and recurrent or metastatic NPC patients, confirmed by pathology and imaging, and without other tumors or history of chemotherapy treatment, 0 < Eastern Cooperative Oncology Group scores < 2; (3) intervention: GP, FP; primary outcomes: 1‐year and 3‐year survival rates, remission rate; second outcomes: neutropenia rate, reduced hemoglobin rate, reduced platelet rate, and digestive symptoms rate. The exclusion criteria were: duplicate, review, letter, comment, and study with uncorrelated or insufficient data.

### 2.3 Data extraction and quality assessment

Two investigators independently extracted data using standard Excel sheets. Any discrepancy was resolved by discussion and consensus. The following information was extracted from each included study: first author, year of publication, GP and FP regimens, pathology type, sample size, and outcomes (mentioned in inclusion criteria). Potential studies were assessed using the Cochrane risk of bias scale,^9^ which consisted of 7 items: random sequence generation; allocation concealment; blinding of participants and personnel to the study protocol; blinding of outcome assessment; incomplete outcome data; selective reporting; other bias. Each item had 3 options: high risk, low risk, and unclear risk. A study was considered as high risk as long as one item was assigned as high‐risk bias.

### 2.4 Statistical analysis

Relative risks (RRs) with 95% confidence intervals (CIs) were calculated to evaluate the efficacy and adverse effects of 1 regimen in treatment of advanced NPC. Heterogeneity within studies was assessed using the *Q* Chi‐square test and *I* ^2^ statistic.^10^ In case of significant heterogeneity (*I* ^2^ > 50% or *P* < .05), a random‐effect model was used for dichotomous outcomes; otherwise, a fixed‐effect model was used.^11^ Sensitivity analysis was also conducted to assess the stability of pooled results. Publication bias was evaluated by observing a funnel plot and confirmed by using Egger and Begg tests.^12^, ^13^ Statistical analyses were conducted on Stata 14.0 (Stata Corp. LP) and RevMan 5.3, with significant level at *P *<* *.05.

## 3 RESULTS

### 3.1 Trial selection

**Figure 1:**
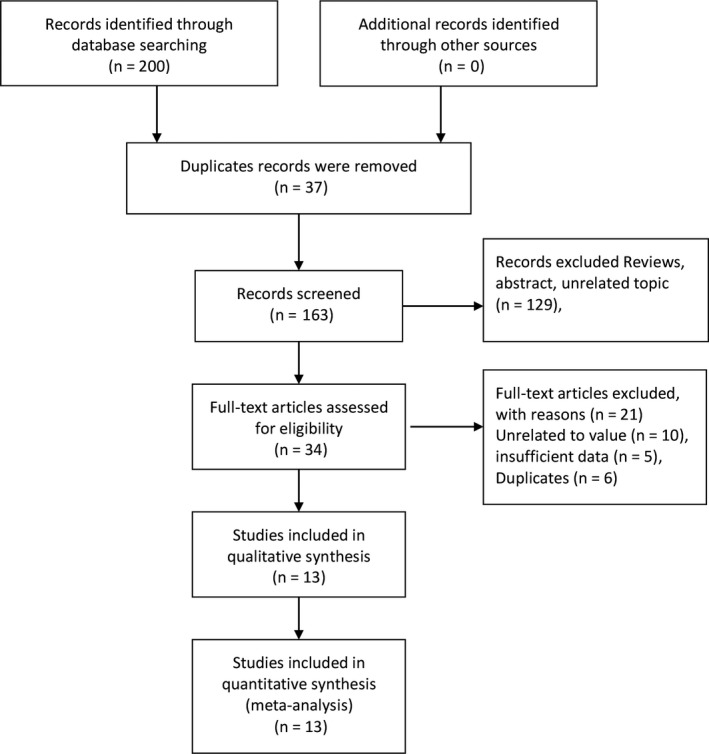
Flowchart of study selection

Figure 1 presents the process of study screening and selection. Our initial search returned 200 records. After duplicates were removed, 163 records were sent to further screening, which excluded 129 records because of review or unrelated topic. Of the remaining 34 studies, full‐text assessment excluded 21 studies because of unrelated value (n = 10), insufficient data (n = 5) and duplicates (n = 6). Finally, 13 studies entered final qualitative synthesis and quantitative analysis.^14^, ^15^, ^16^, ^17^, ^18^, ^19^, ^20^, ^21^, ^22^, ^23^, ^24^, ^25^, ^26^

### 3.2 General characteristics and quality assessment

The general characteristics of the included studies were presented in Table 1. These studies were published between 2006 and 2016 and their sample sizes ranged from 42 to 362. Almost all cases were pathologically confirmed to be NPC. The GP regimen was Gem +DDP, and the FP regimen was 5‐Fu +DDP. The observed outcomes included 1‐year and 3‐year survival rates, remission rate, neutropenia, reduced hemoglobin, reduced platelet, and digestive symptoms. One study only reported 3‐year survival rate, and one study only reported remission rate. Supplements S1 and S2 list the investigators’ judgments about each risk of bias item presented as percentages across all included studies, and about each risk of bias item for each included study, respectively. Overall, 8, 2 and 3 studies were categorized as low, unclear and high‐risk bias, respectively. The randomized sequence was adequately reported in 10 studies, appropriate allocation concealment was reported in 8 studies, but blinding application was unclear in most studies.

**Table 1:** General characteristic of included randomized controlled trials in the meta‐analysis

Author	Year of publication	GP/FP	Pathology	Sample size		Outcomes
				GP	FP	
Bai et al	2006	Gem 200 mg·m^−2^5‐Fu 500 mg·m^−2^d1‐5 + DDP25 mg·m^−2^d1‐5	Squamous carcinoma	93	94	1‐y survival rate, 3‐y survival rate, remission rate, neutropenia, reduced hemoglobin, reduced platelet, digestive symptoms
Cai et al	2009	Gem 1000 mg·m^−2^d1, 8 + DDP25 mg·m^−2^d1‐45‐Fu 500 mg·m^−2^d1‐5 + DDP25 mg·m^−2^d1‐4	Squamous carcinoma	29	32	1‐y survival rate, 3‐y survival rate, remission rate, reduced hemoglobin, reduced platelet, digestive symptoms
Hu et al	2012	Gem 1000 mg·m^−2^d1, 8 + DDP25 mg·m^−2^d1‐45‐Fu 500 mg·m^−2^d1‐5 + DDP25 mg·m^−2^d1‐4	Squamous carcinoma	33	33	1‐y survival rate, remission rate, reduced hemoglobin, reduced platelet
Jin et al	2012	Gem 1000 mg·m^−2^d1, 8 + DDP25 mg·m^−2^d1‐35‐Fu 1000 mg·m^−2^d1‐5 + DDP80 mg·m^−2^d1‐3	–	173	176	1‐y survival rate, 3‐y survival rate, remission rate, neutropenia, reduced hemoglobin, reduced platelet, digestive symptoms
Mo et al	2010	Gem 1000 mg·m^−2^d1, 8 + DDP25 mg·m^−2^d1‐35‐Fu 500 mg·m^−2^d1‐5 + DDP25 mg·m^−2^d1‐3	Squamous carcinoma	27	29	Remission rate, reduced hemoglobin, digestive symptoms, mucosal inflammation
Yan et al	2006	Gem 1250 mg·m^−2^d1, 8 + DDP25 mg·m^−2^d15‐Fu 1000 mg·m^−2^d1‐5 + DDP25 mg·m^−2^d1	Squamous carcinoma	34	28	1‐y survival rate, 3‐y survival rate, remission rate, neutropenia, reduced hemoglobin, reduced platelet, digestive symptoms
Wang et al	2015	Gem 1000 mg·m^−2^d1, 8 + DDP25 mg·m^−2^d1‐35‐Fu 500 mg·m^−2^d1‐5 + DDP25 mg·m^−2^d1‐3	–	30	16	Remission rate, mucosal inflammation
Yuan et al	2009	Gem 1000 mg·m^−2^d1, 8 + DDP25 mg·m^−2^d1‐45‐Fu 400 mg·m^−2^d1‐5 + DDP25 mg·m^−2^d1‐4	Squamous carcinoma	30	30	Remission rate, neutropenia, reduced hemoglobin, reduced platelet, digestive symptoms, mucosal inflammation
Zhao et al	2010	Gem 1000 mg·m^−2^d1, 8 + DDP25 mg·m^−2^d1‐35‐Fu 100 mg·m^−2^d1‐5 + DDP25 mg·m^−2^d1‐3	Squamous carcinoma	23	19	Remission rate, neutropenia, reduced hemoglobin, reduced platelet, digestive symptoms, mucosal inflammation
Zou et al	2011	Gem 1000 mg·m^−2^d1,8 + DDP25 mg·m^−2^d1‐45‐Fu 75 mg·m^−2^d1‐5 + DDP75 mg·m^−2^d1‐5	Squamous carcinoma	26	28	1‐y survival rate, 3‐y survival rate, remission rate, neutropenia, reduced hemoglobin, reduced platelet, digestive symptoms
Gu et al	2012	Gem 800 mg·m^−2^d1,8 + DDP20 mg·m^−2^d1‐45‐Fu 500 mg·m^−2^d1‐5 + DDP20 mg·m^−2^d1‐4	Squamous carcinoma	80	80	3‐y survival rate, neutropenia, reduced hemoglobin, reduced platelet, digestive symptoms
Zheng et al	2015	Gem 1000 mg·m^−2^d1, 8 + DDP25 mg·m^−2^d1‐35‐Fu 800 mg·m^−2^d1‐5 + DDP80 mg·m^−2^d1‐3	Squamous carcinoma	13	36	3‐y survival rate
Zhang et al	2016	Gem1 g·m^2^‐8 cisplatin 80 mg·m^2^‐15‐Fu 4 g·m^2^ 96 h cisplatin 80 mg·m^2^‐1	Squamous carcinoma	181	181	Anemia, neutropenia, digestive symptoms

### 3.3 Primary outcomes

**Figure 2:**
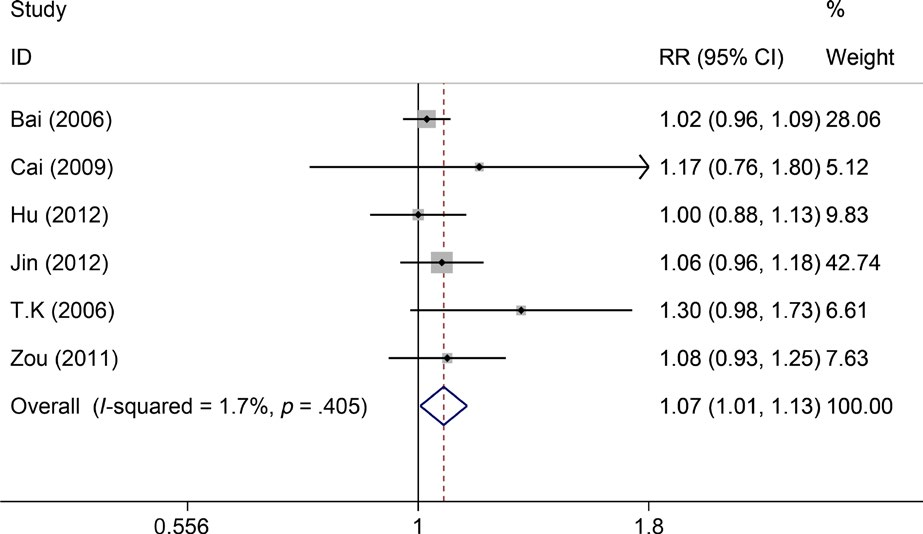
Comparisons of 1‐year survival rate between GP and FP

**Figure 3:**
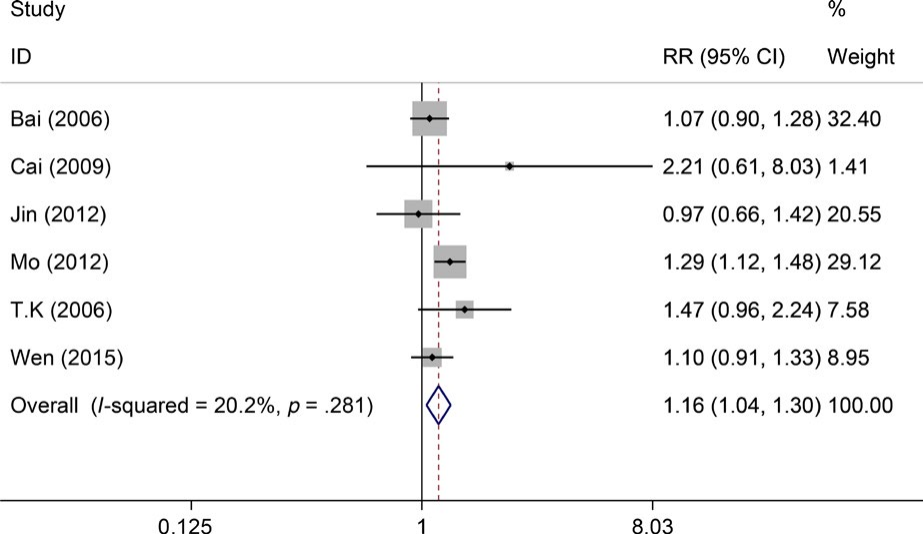
Comparisons of 3‐year local control between GP and FP

**Figure 4:**
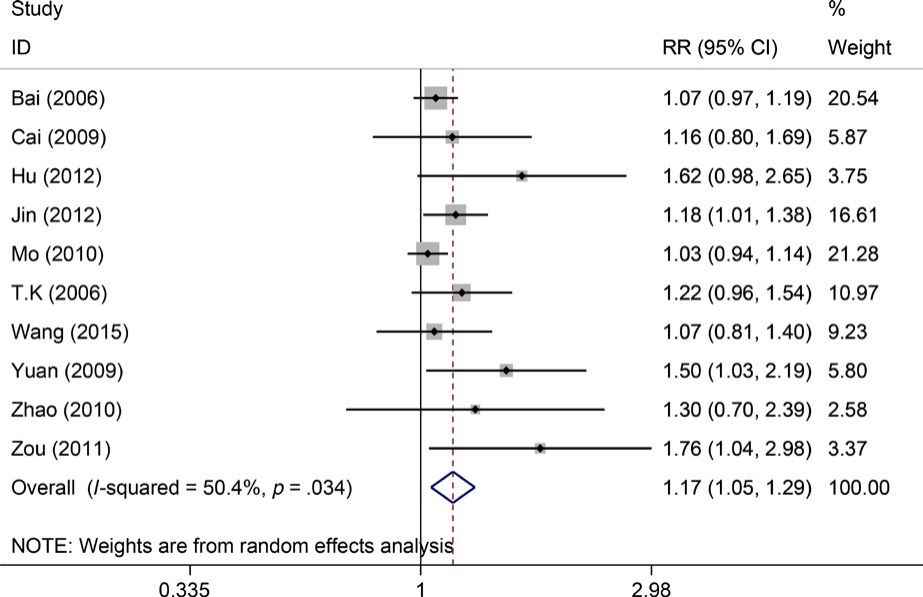
Comparisons of remission rate between GP and FP

Six studies reported 1‐year survival rate, but no significant heterogeneity across studies was found (*I* ^2^ *= *1.7%, *P *=* *.405). Thus, the fixed‐effect model was used, which showed the GP regimen significantly increased the 1‐year survival rate compared with the FP regimen (RR = 1.07, 95%CI: 1.01‐1.13, *P* = .033, Figure 2). Also, 6 studies reported 3‐year survival rate, but the heterogeneity within studies was low (*I* ^2^ *= *20.2%, *P *=* *.281). Thus, the fixed‐effect model was used, which indicated the GP regimen significantly outperformed the FP regimen (RR = 1.16, 95%CI: 1.04‐1.30, *P *=* *.007, Figure 3). Ten studies reported the remission status but were found with moderate heterogeneity (*I* ^2^ *= *50.4%, *P *=* *.034). Thus, the random‐effect model was used, which indicated the remission rate of the GP regimen was significantly higher than the FP regimen (RR = 1.17, 95%CI: 1.05‐1.29, *P *<* *.001, Figure 4).

### 3.4 Second outcomes

Ten studies reported severe adverse gastrointestinal effects, and the heterogeneity within studies was medium (*I* ^2^ *= *46.1%, *P *=* *.054). No significant difference was found in this outcome (RR = 0.58, 95%CI: 0.45‐0.74, *P *<* *.001). Eight studies gave data about reduced hemoglobin rate and were found with high heterogeneity (*I* ^2^ *= *58%, *P *=* *.042). Thus, the random‐effect model was used, which showed no significant difference in reduced hemoglobin rate (RR = 0.55, 95%CI: 0.36‐1.21, *P *=* *.464). Eleven studies provided information about neutropenia. The fixed‐effect model indicated no significant difference (*I* ^2^ =* *32%, *P *=* *.178; RR = 1.84, 95%CI: 0.93‐5.02, *P *=* *.512). Nine studies reported reduced platelet, but no significant difference was found (RR = 1.25, 95%CI: 0.85‐1.75, *P *=* *.406). Results from 5 studies indicated that there was no significance in mucosal inflammation between 2 treatment plans (RR = 0.81, 95%CI: 0.57‐1.16).

### 3.5 Sensitivity analysis and publication bias

**Figure 5:**
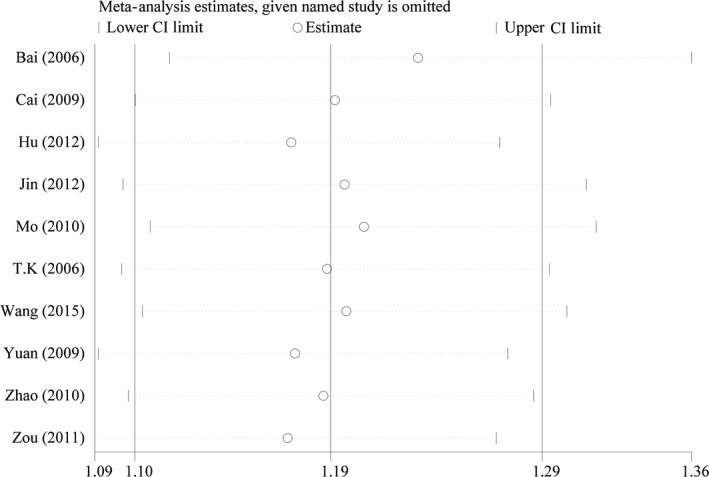
Sensitivity analysis of remission rate comparisons

**Figure 6:**
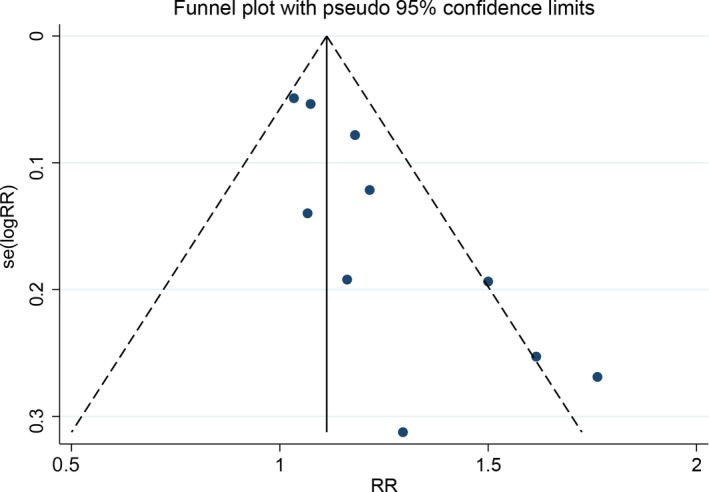
Detection of publication bias

The remission rate consisting of more number of study was used to conduct sensitivity analysis, which indicated the results ranged from 1.10 to 1.29 (Figure 5). The funnel plot showed asymmetry in the lower segments, in which small negative trials were missing (Figure 6). The Begg and Egger tests indicated the potential presence of some publication bias (*Z* = 1.970, *P *=* *.049; *t* = 4.060, *P *=* *.004).

## 4 DISCUSSION

Our study indicates the GP regimen can better improve the 1‐year and 3‐year survival rates of advanced NPC patients compared with the FP regimen. The remission rate in the GP regimen is higher than in the FP regimen. The adverse reactions are not significantly different between regimens. Distant metastasis is an important factor influencing the prognosis of advanced NPC patients. It has become urgent to improve the survival status of advanced NPC patients. Different from other head and neck tumors, advanced NPC is well sensitive to chemotherapy. The most commonly‐used single chemotherapy agents are bodeomycin, methotrexate, 5‐fluorouracil, cisplatin and carboplatin, and the effective rates are almost 20%. Our results indicate the gemcitabine + cisplatin regimen facilitates the prognosis of advanced NPC patients.

Gemcitabine is a cytarabine analogue that exerts antitumor effect mainly by affecting the S phase and late G1 phase of DNA synthesis. Gemcitabine can intervene DNA repair mechanisms through the unique masking chain and lead to cell apoptosis.^27^, ^28^ At present, gemcitabine is mainly used for nonsmall cell lung cancer, advanced pancreatic cancer and other cancers, brining satisfactory curative effects with low toxicity during tumor treatment.^29^ Gemcitabine has no cross‐resistance with PF chemotherapy in early NPC patients, and is appropriate for NPC patients with or without receiving PF chemotherapy. The 2008 National Comprehensive Cancer Network also recommended gemcitabine for patients with advanced NPC and recurrence or distant metastasis who cannot receive surgical removal of recurrence or distant metastasis salvage treatment. The combined application of Gisitama and DPP has a synergistic or superimposed effect. As reported, the complete remission rate is 42.7%, 1‐year survival rate is 33.9%, the median progression‐free survival and overall survival are 5. 6 and 9 months with little III‐IV degree adverse reaction, respectively.^30^ These results support the superiority over the PF regimen and are consistent with our results. However, the difference is that we did not observe any significant difference in adverse effects between GP and FP regimens. Gu et al^24^ reported no significant differences in 3‐year overall survival rate or disease‐free survival rate between the GP and PF regimens. This study with 240 patients found the overall survival rates of 2 regimens were both 95% but reported no differences in toxicity.^24^ On the contrary, Zheng reported that induction chemotherapy had no survival benefit, but the GP regimen benefited overall survival and tended to improve distant‐ metastasis‐free survival of locoregionally advanced NPC patients.^25^ The GP regimen was an independent prognostic factor for overall survival and trended to improve distant‐metastasis‐free survival, while the TP regimen (taxol + cisplatin) was only a significant prognostic factor for distant‐metastasis‐free survival.^25^ These findings also indicate the GP regimen is superior for locoregionally advanced NPC.

Nevertheless, this meta‐analysis has several limitations. First, all included studies were from published the literatures, and some gray literatures and unpublished data were not included, which may cause some publication bias. Second, since NPC is a typical regional disease mostly occurring in South China and Southeast Asia, the included studies are from Asia, which is one of causes for publication bias. Third, the sample sizes of most studies are quite small, which may reduce the accuracy of findings. Finally, though the types and drugs in the included studies are the same, differences in doses, treatment periods and cycles may cause potential bias.

In conclusion, the GP regimen outperforms the FP regimen in treatment of advanced NPC, but with no difference in adverse effects. We may consider the GP regimen as a better choice, but this conclusion should be verified by high‐quality trials.

## CONFLICT OF INTEREST

None declared.
