# Supplementary figures and images for: Gemcitabine and cisplatin regimen facilitates prognosis of advanced nasopharyngeal carcinoma
Source: Cancer Med. 2018 May 23;7(7):2985–92. doi: 10.1002/cam4.1575 (PMC6051151; doi:10.1002/cam4.1575)

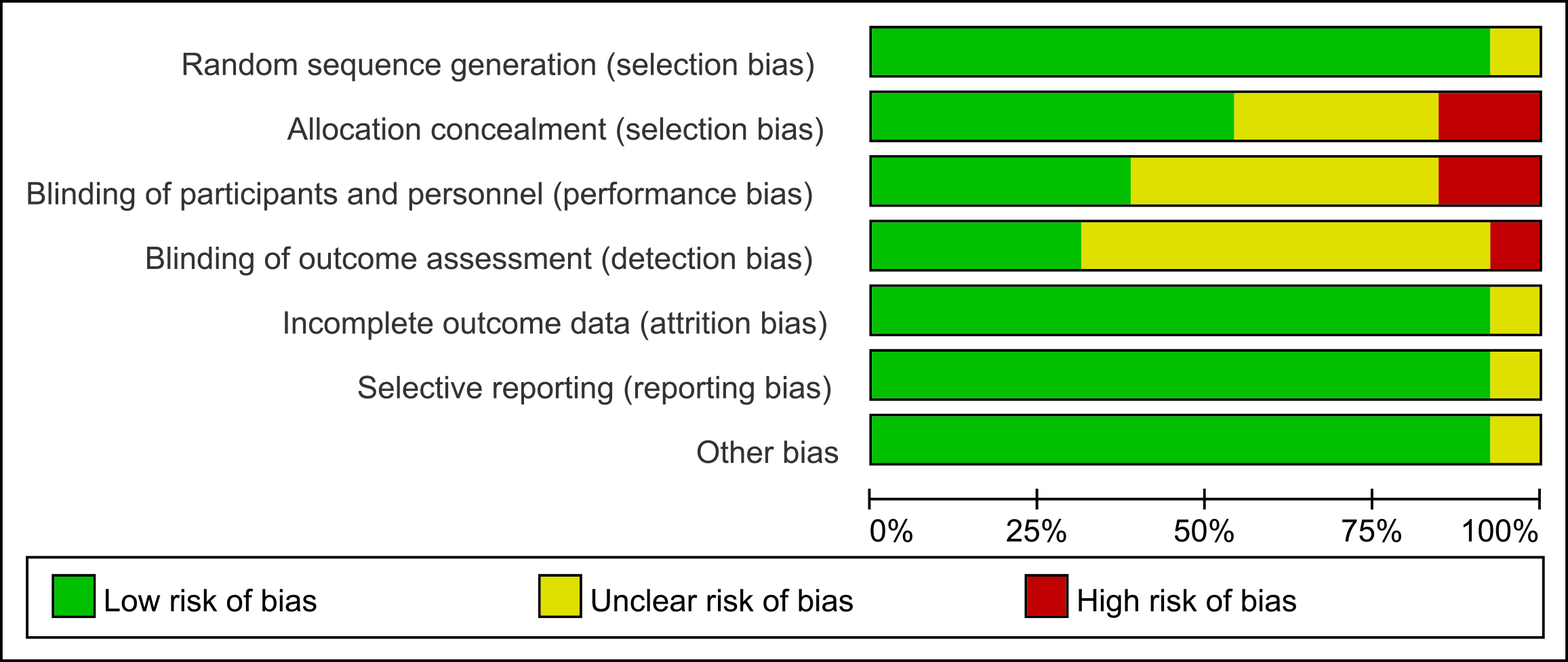

Supplement: Supplementary file 1 [file CAM4-7-2985-s001.tif]

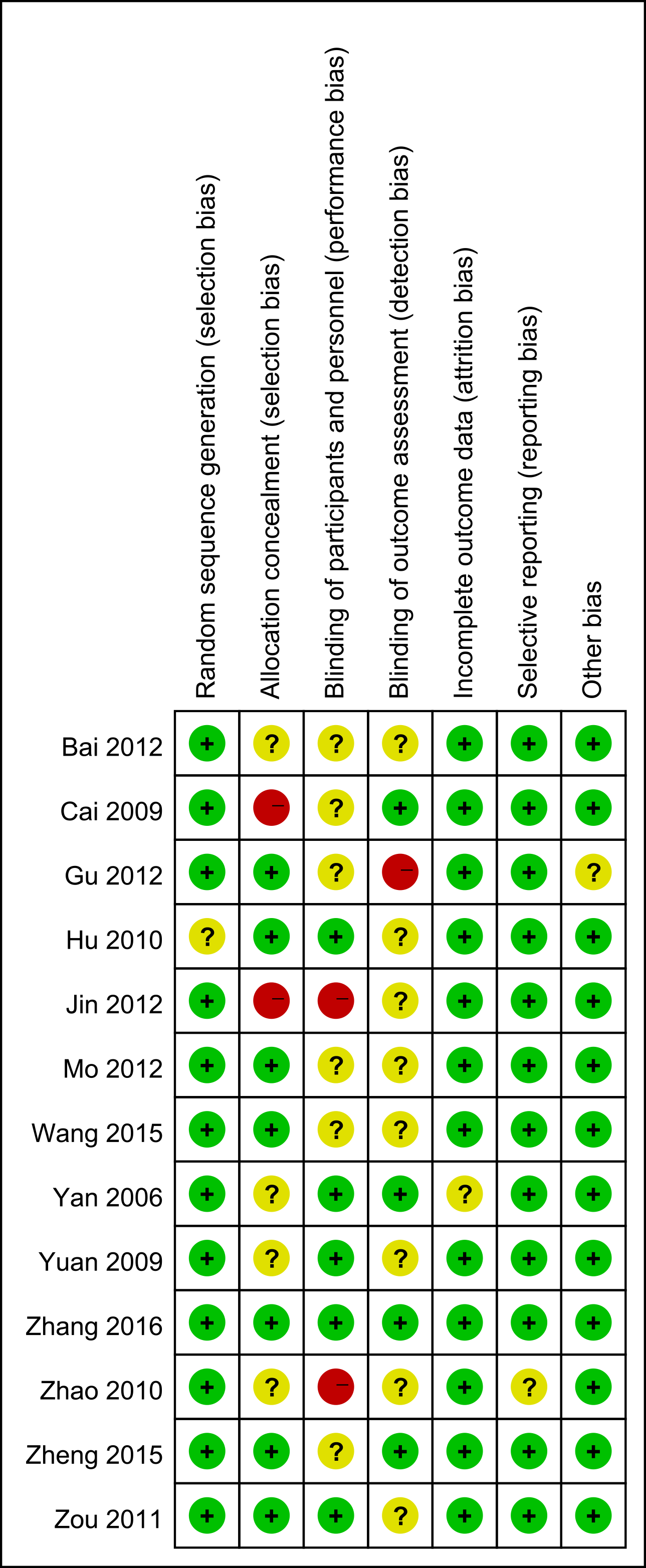

Supplement: Supplementary file 2 [file CAM4-7-2985-s002.tif]
