# Supplementary material for: Gemcitabine and cisplatin regimen facilitates prognosis of advanced nasopharyngeal carcinoma
Source: Cancer Med. 2018 May 23;7(7):2985–92. doi: 10.1002/cam4.1575 (PMC6051151; doi:10.1002/cam4.1575)
Supplement: Supplementary file 3 [file CAM4-7-2985-s003.docx]

**Supplementary materials**

**Supplementary material 1** review authors' judgments about each risk of bias item presented as percentages across all included studies.

**Supplementary material 2** Risk of bias summary: review authors' judgments about each risk of bias item for each included study.
